# Dietary fiber pectin alters the gut microbiota and diminishes the inflammatory immune responses in an experimental peach allergy mouse model

**DOI:** 10.1038/s41598-024-82210-3

**Published:** 2024-12-16

**Authors:** Hanna Steigerwald, Melanie Albrecht, Birgit Blissenbach, Maren Krause, Andrea Wangorsch, Maike Schott, Irene Gonzalez-Menendez, Leticia Quintanilla-Martinez, Masako Toda, Stefan Vieths, Oleg Krut, Stephan Scheurer, Frank Blanco-Pérez

**Affiliations:** 1 Molecular Allergology, Paul-Ehrlich-Institut, Federal Institute for Vaccines and Biomedicines, Langen, Germany; 2 Microbiological Safety, Paul-Ehrlich-Institut, Federal Institute for Vaccines and Biomedicines, Langen, Germany; 3 Institute of Pathology and Neuropathology, Comprehensive Cancer Center, University Hospital Tübingen, Eberhard Karls University of Tübingen, Tübingen, Germany; 4 Cluster of Excellence iFIT (EXC 2180) “Image-Guided and Functionally Instructed Tumor Therapies”, Eberhard-Karls University of Tübingen, Tübingen, Germany; 5 Laboratory of Food and Biomolecular Science, Graduate School of Agricultural Science, Tohoku University, Sendai, Miyagi 980-8577 Japan

**Keywords:** Dietary fiber, Pectin, Peach allergy, Immune modulation, Gut microbiota, Dietary carbohydrates, Immunology, Immunological disorders, Gastrointestinal diseases, Immunological disorders, Nutrition disorders, Experimental models of disease, Preclinical research

## Abstract

Since therapeutic options are limited the utilization of prebiotics is suggested to prevent food allergies (FAs). Using an experimental peach allergy model we explored the effect of dietary fiber pectin, a high-methoxyl heteropolysaccharide, on the manifestation of FA. CBA/J mice were sensitized, subsequently orally boosted and provoked with peach peel extract. For dietary intervention, mice were fed a pectin containing diet before (primary-preventive) or after (secondary-preventive) sensitization. Non-treated allergic and sham-treated mice were fed a diet containing 20% cellulose. Fecal microbiota, humoral and intestinal immune cell responses were analyzed. Pectin remarkably affected the gut microbiota composition and diversity, promoting mainly the growth of *Bacteroides*. The frequency of mast cells, macrophages, and CD3^+^T cells in the lamina propria of the small intestine was reduced, whereas the frequency of B cells and CD4^+^T cell subpopulation was enhanced. Pectin intervention in the primary-preventive stetting significantly triggered serum IgA levels, whereas production of IgE and mMCPT-1 was reduced. Remarkably, in both settings peach allergen-specific IgG1/IgG2a ratio and specific IgE were significantly reduced to baseline. The data suggest, that dietary supplementation of pectin in both intervention approaches can diminish inflammatory responses and signs of allergic immune responses, accompanied by alteration of the gut microbiota composition.

## Introduction

Food allergy (FA) is a potentially life-threatening immune reaction with increasing prevalence worldwide^1^. FAs are typically IgE-mediated type I hypersensitivity reactions, characterized by a type 2 (T2) inflammation accompanied by the induction of T helper type 2 (T_H_2) cells. The activation of mast cells and basophils leads to local inflammatory and systemic reactions which may even accumulate to life-threatening anaphylactic reactions^2^. Peach allergy is a very frequent fruit allergy in Japan and especially in the Mediterranean area. The clinical manifestation can range from mild oral symptoms to anaphylaxis depending on the geographical distribution and the sensitization profile^3^. Remarkably, the reason for the geographical restriction is still unknown and immunotherapeutic strategies are not available.

Different therapeutic options such as oral immunotherapy (OIT) or sublingual immunotherapy (SLIT), which aimed to induce desensitization or peripheral tolerance, showed promising results in a limited number of studies^4–6^. So far, the only licensed product for OIT of food allergy has been approved by the US Food and Drug Administration for treatment of peanut allergy^7^. Thus, the recommended management of FAs is still symptomatic treatment, the avoidance of the allergenic food or a healthy diet, e.g. using prebiotics^8^.

Since the dysbiosis of gastrointestinal (GI) microbiota has been recently associated as a main risk factor for development of allergic airway inflammation and FA^9–11^ targeting the GI-microbiota to retain homeostasis has been discussed as preventive option for FA^12–14^. Particularly, dietary fibers, which are not digested by GI-enzymes but can be degraded by commensal bacteria in the colon, gained attention due to their ability to affect GI-microbiota composition, avoiding dysregulation, and promoting production of bacteria-derived metabolites with immune-modulating properties^15–17^. In line with this, the dietary fiber pectin has been reported to promote several additional health benefits as the improvement of physical bowel function, maintenance of blood cholesterol and glycemic response^15,18–20^. Pectin is a dietary fiber which promotes the growth of health-promoting bacteria^21^. Bacterial fermentation of pectin, like dietary fibers, is well known to release different metabolic products like short chain fatty acids (SCFAs), including acetate, propionate and butyrate which mediate immune-modulatory effects^22–24^. Two health claims have been accepted for pectin by the EU: 1^st^ reduction of the blood glucose rise after meals and 2^nd^ maintenance of normal blood cholesterol levels after consumption of at least 6–10 g pectin per meal^25^. Early studies on allergic asthma revealed beneficial effects of pectin supplementation in mice due to modulation of the microbiota, with increased levels of circulating SCFAs and protection against the development of allergic inflammation in the lung^26,27^. However, reports on the potential prophylactic and therapeutic effects of pectin in experimental food allergy models and the associated effects on the GI-immune response in a T_H_2-biased allergic setting are limited.

Previous own and independent studies showed that the chemical structure of pectin, which is characterized by the molecular mass, carbohydrate composition of the backbone and branches, degree of esterification and blockiness, impacts its fermentation efficacy^22,28–30^. Data provide evidence that pectin with high degree of methyl-esterification (HMP) exerts strongest effects on the immune response in naïve mice^28,30^. Therefore, the present study aimed to investigate the effect of dietary intervention with HMP derived from apple on the development of clinical signs, inflammation, as well as local and humoral immune responses in a peach allergy mouse model.

## Results

### Pectin supplementation did not prevent the development of allergy-related clinical signs

**Fig. 1:**
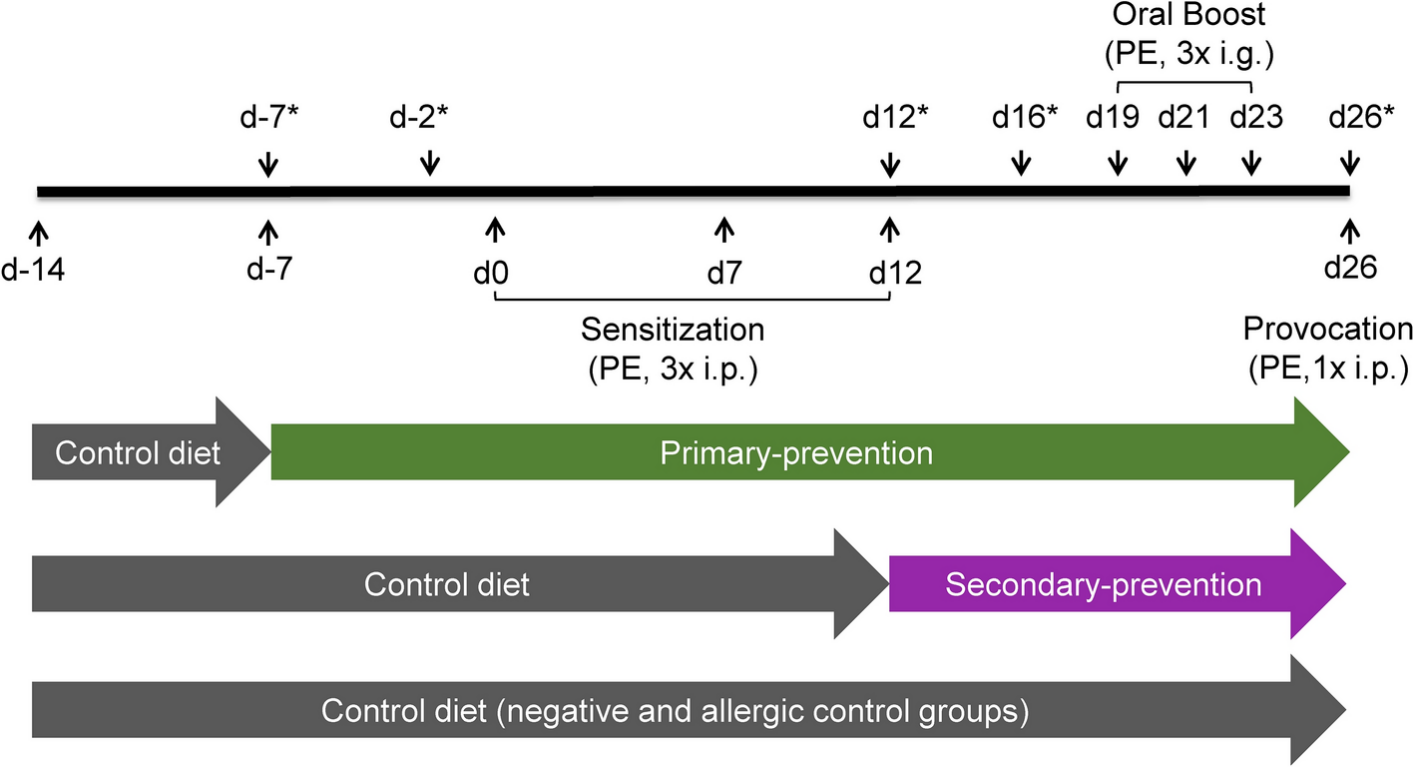
Experimental schedule of pectin intervention in peach allergic mice. Mice were sensitized intraperitoneally (i.p.) using peach peel extract (PE) three times (d0, d7 and d12), followed by oral (i.g.) boost of PE three times and finally mice were subjected to provocation with i.p. injection of PE. For primary-preventive pectin intervention, pectin feeding (15% HMP plus 5% cellulose) started seven days before sensitization. Secondary-preventive intervention started after sensitization of the mice. PBS and allergic controls were fed control diet (20% cellulose) during the course of the experiment. Feces samples were taken at indicated time points (*).

**Fig. 2:**
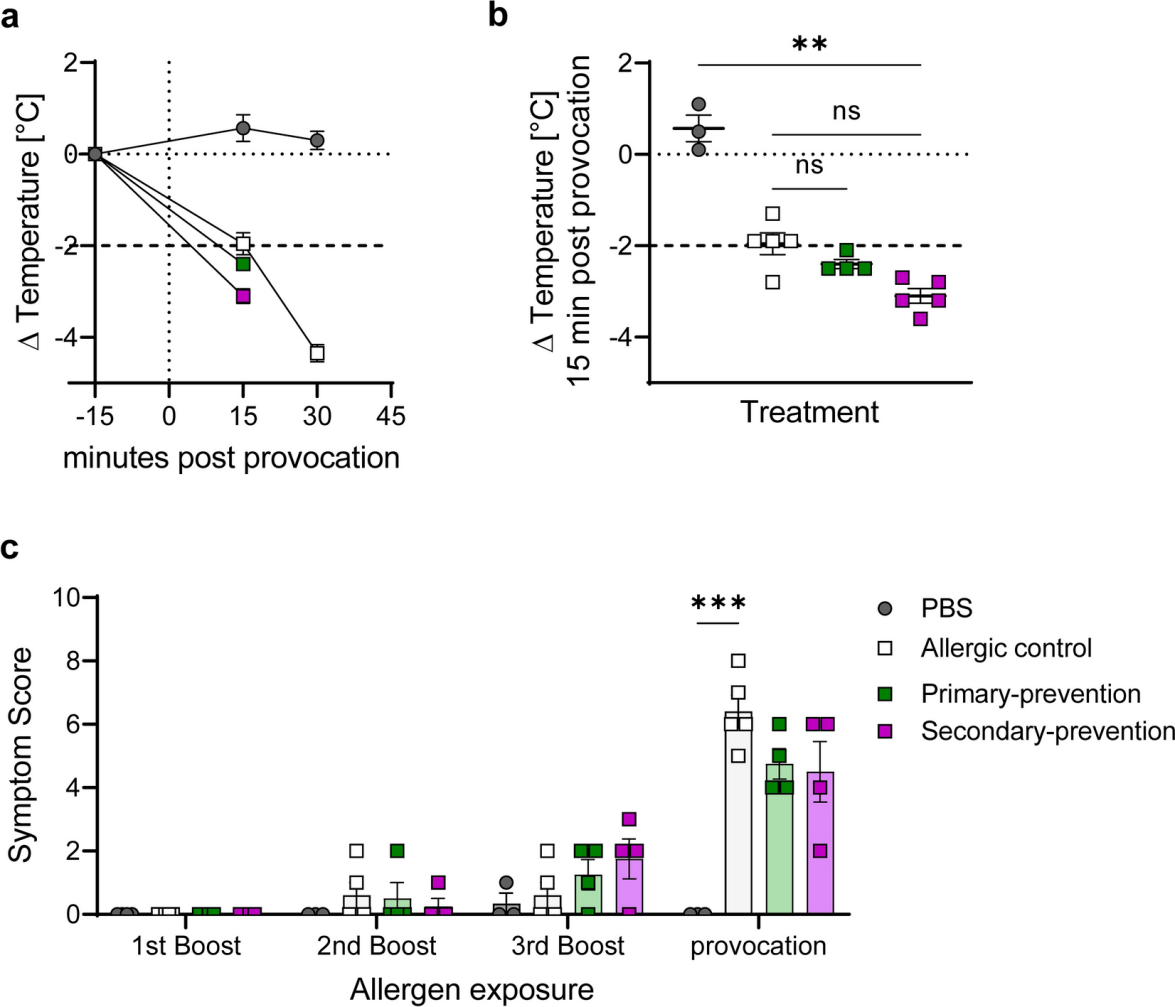
Allergy-related signs and symptoms after provocation. (**a**) Body temperature was measured before (-15 min; baseline) and up to 30 min after i.p.-provocation. (**b**) Body temperature of individual mice 15 min after i.p.-provocation and (**c**) symptom score after oral exposition and provocation. n = 3–5; *p < 0.05; **p < 0.01; ***p < 0.001, one-way ANOVA and two-way ANOVA were used in b and c, respectively.

Two different intervention approaches, a primary-preventive (prophylactic) and secondary-preventive (therapeutic) treatment strategy, were compared in a mouse model of peach allergy (Fig. 1). Monitoring of body weight gain and food intake after the course of the experiments (26 days) did not reveal significant differences between the treatment groups and between treatment and control groups (Figure S1). Body core temperature was monitored up to 30 min after provocation with peach extract (PE) at day 26 (Fig. 2a). A significant temperature drop could be observed 15 min after the provocation in all PE exposed groups in comparison to the non-sensitized PBS control. However, the results did not show any difference between both, pectin treated mice and the allergic control (Fig. 2b). Noteworthy, mice with a temperature drop of more than 2 °C were sacrificed according to the animal guidelines. In addition, allergy-related clinical signs were examined in a blinded manner, including behavioral changes, consistency of the stool as well as ruffled fur (Table S1). For all PE-sensitized groups (with and without pectin treatment) mild allergy-related signs were observed after the 2nd oral boost, slightly increasing by the 3rd boost (Fig. 2c). The strongest symptoms were observed after systemic provocation with PE, demonstrating the allergy model was successfully established. However, only a slight reduction of the overall symptom score could be observed in the groups supplemented with pectin compared to the allergic control.

### Pectin consumption altered size of intestine and gut microbiota composition in peach allergic mice

The effect of pectin intervention on both, the intestinal tissue and gut microbiota composition was evaluated.

The length of different intestinal sections (small and large intestine and caecum) was measured and histological evaluation was performed (Figure S2a–c). Results showed that the length of the small intestine was significantly reduced in the primary-preventive pectin group (Figure S2a), whereas the length of the large intestine and caecum were significantly enlarged in both pectin intervention groups when compared to the allergic control (Figure S2b,c).

**Fig. 3:**
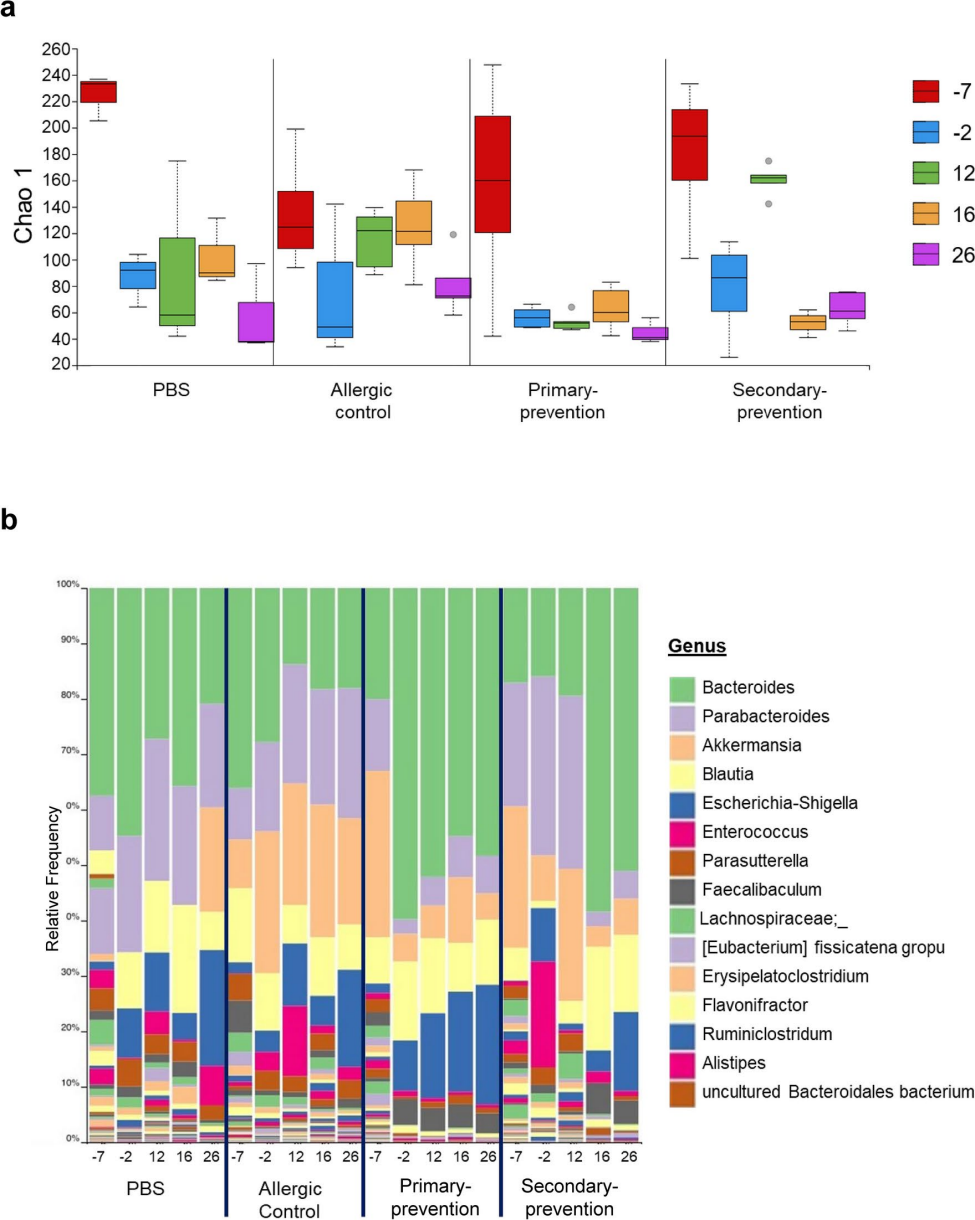
Alteration of gut microbiota composition. (**a**) Microbiota diversity (Chao 1) and (**b**) relative frequency of bacterial genera was analyzed in feces of the mice of the four treatment groups at different time points (d-7, d-2, d12, d16, d26).

To determine, whether pectin intervention induced alterations of the gut microbiota composition, feces samples were collected at different time points (as indicated in Fig. 1) and analyzed by metagenomics 16S rRNA gene sequencing (Fig. 3). The standard cellulose diet (in both control groups and secondary-preventive group) resulted in a reduction in microbial richness as indicated by the Chao 1 index at d-2 compared to d-7. (Fig. 3a).

Pectin intervention led to a more prominent and persistent reduction of the microbial richness, especially in the primary-preventive approach 5 days after starting pectin exposure (at d-2) and in the therapeutic approach 4 days after starting the pectin exposure (at d16). In contrast, data provide evidence that allergic sensitization (from d0 to d12) without the simultaneous exposure to pectin promoted the microbial richness in the allergic control group and the secondary-preventive group.

Above changes in the microbiome complexity correlated with a substantial shift of the bacterial composition towards *Bacteroides spp.*, which was observed already 4–5 days after start of the pectin diet (d-2 for primary-preventive, d16 for secondary-preventive) (Fig. 3b; S3). In contrast to the increase of *Bacteroides spp.* in response to the pectin intervention, the relative frequency of bacteria belonging to this genus seemed to be reduced in the mice of the allergic control after allergic sensitization (d12). Moreover, the frequency of *Parabacteroides spp.* decreased after pectin treatment when compared to the allergic control, and the frequency of *Akkermansia spp.* was strongly reduced after start of pectin intervention, whereas the levels stayed constant in the allergic control group (Figure S3). Noteworthy, the pectin induced change of the composition of microbiota was consistent for all individual mice of a group (Figure S4). In contrast, the relative frequency of genus *Blautia* slightly increased after introduction of pectin in both treatment groups (d16 vs d12 in the secondary-preventive and d-2 vs d-7 in the primary-preventive group) (Table S4).

### Pectin intervention modulated the local immune response in the intestinal tract

Considering the changes observed in the intestine size and gut microbiota after pectin intervention, the possible effects on immune cells in the lamina propria were analyzed by histology and FACS.

Histological analysis of the small intestine revealed slightly dilated lumen of the allergic control group, with no significant impact on inflammatory infiltration. Both pectin intervention groups showed dilated lumen, slightly shorter villi and minimal inflammatory infiltrate. Mild apical edema was observed in the secondary prevention group. No strong histological differences between the treatment groups could be observed. Other histopathological alterations as hypertrophic goblet cells, hypertrophic Paneth or thicker muscle layer were not detected in any sample (Figure S5).

**Fig. 4:**
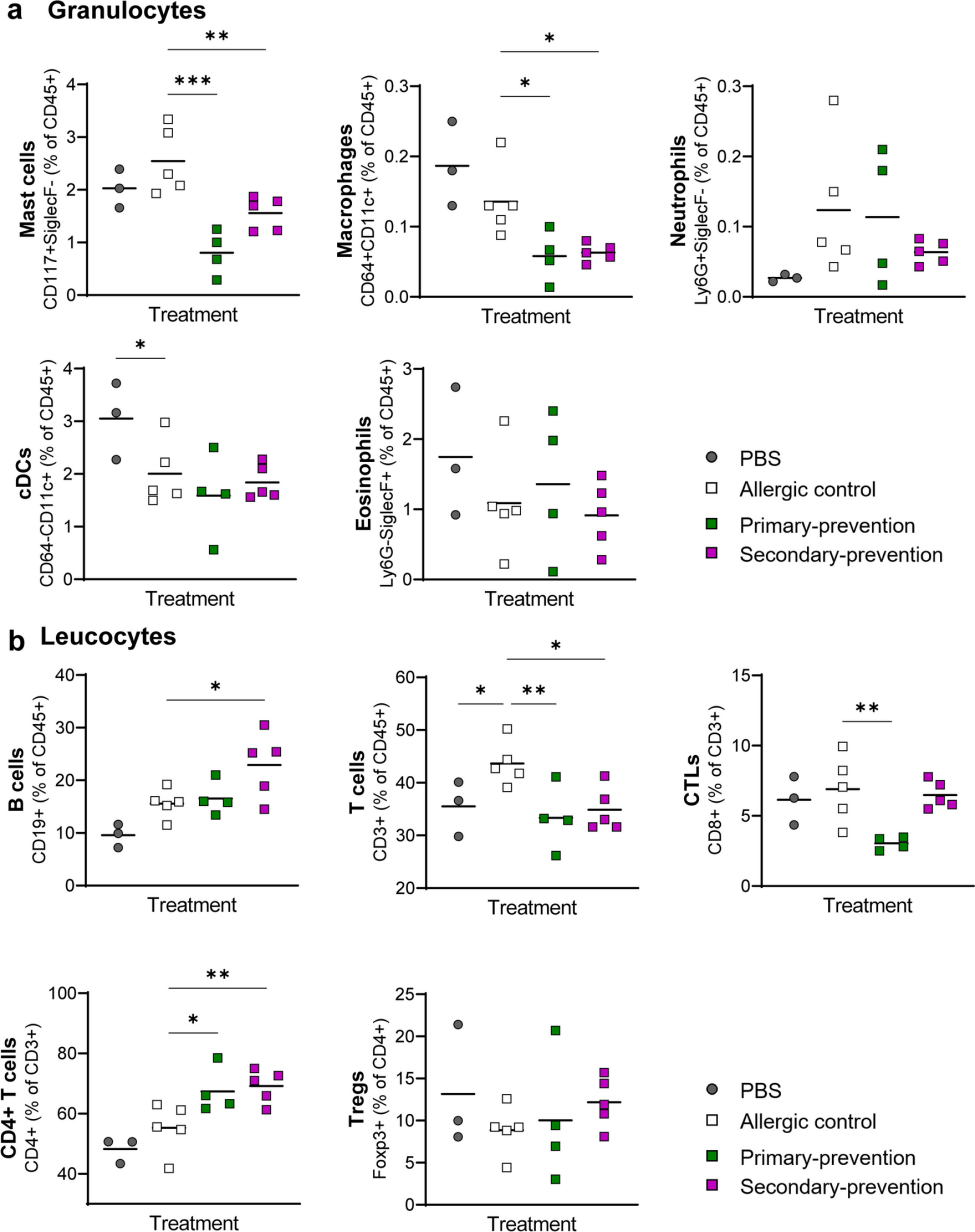
Frequency of immune cells in the small intestine lamina propria. The small intestine was enzymatically treated and isolated lamina propria immune cells were analyzed via flow cytometry. Granulocytes (**a**): mast cells (CD45^+^CD117^+^), macrophages (CD45^+^CD11c^+^), neutrophils (CD45^+^Ly6G^+^), cDCs (CD45^+^CD11c^+^) and eosinophils (CD45^+^Siglec F^+^). Leucocytes (**b**): B cells (CD45^+^CD19^+^), T cells (CD45^+^CD3^+^), CTLs (CD45^+^CD3^+^CD8^+^), CD4 + T cells (CD45^+^CD3^+^CD4^+^) and Tregs (CD45^+^CD3^+^CD4^+^Foxp3^+^). n = 3–5; *p < 0.05; **p < 0.01; ***p < 0.001, one-way ANOVA was used.

FACS analysis of the cells from GI tissue showed a significant decrease of levels of mast cells and macrophages in both pectin intervention groups, compared to the allergic control (Fig. 4a). No clear differences were observed in the frequency of neutrophils, cDCs or eosinophils. Interestingly, when mice received pectin upon sensitization (secondary-preventive approach), pectin induced increased levels of B cells (Fig. 4b) and reduced the high frequency of T cells in the allergic setting back to normal levels of non-allergic mice. Further evaluation of T cell subpopulations revealed that pectin modulated differently the T cell subtypes. An enhanced frequency of CD4^+^T cells by both approaches in comparison with the allergic control was observed. In contrast, only primary-preventive pectin intervention showed a reduction on CD8^+^CTLs while no effect on the amount of Tregs (CD4^+^Fox p 3^+^) could be observed between the treatment groups.

### Pectin intervention affects mMCPT-1 secretion

**Fig. 5:**
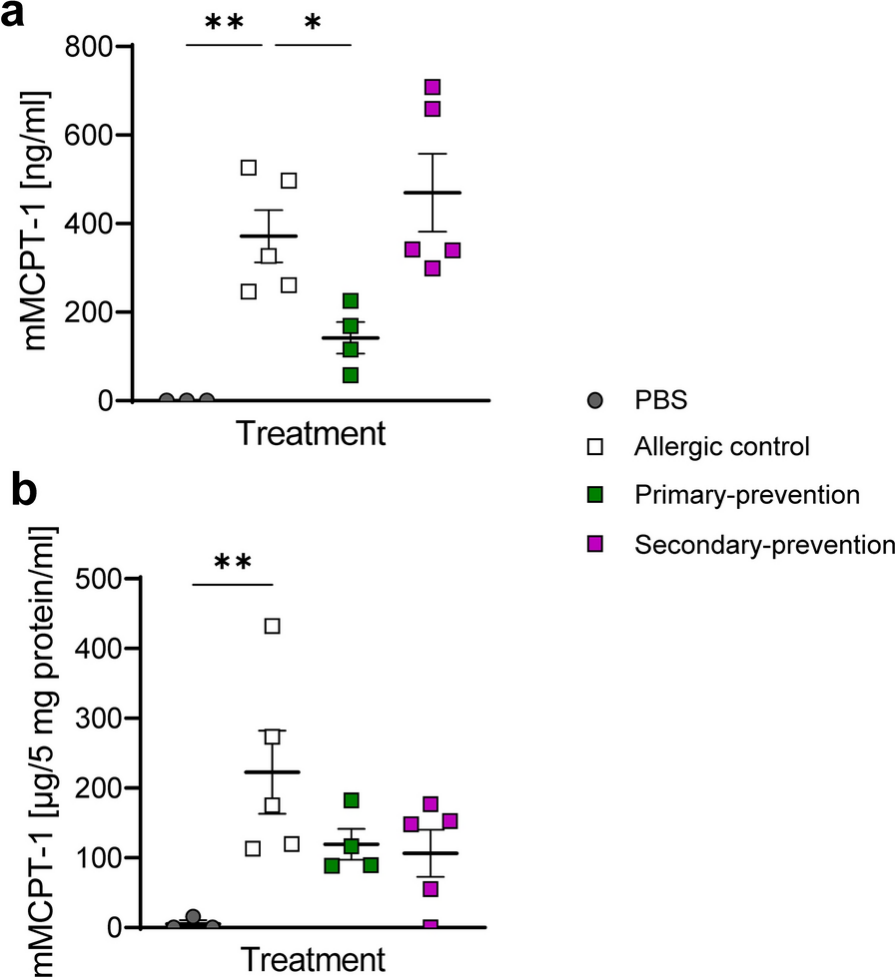
Determination of humoral and local mMCPT-1 levels upon pectin intervention. Levels of mMCPT-1 were measured in (**a**) serum [mMCPT-1 µg/ml] and (**b**) the small intestine homogenates [mMCPT-1 µg/5 mg protein/ml] via ELISA. n = 3–5; *p < 0.05, **p < 0.01, one-way ANOVA was used.

Next, the levels of mouse mast cell protease-1 (mMCPT-1), a mast cell activation marker^31^ were analyzed in serum (Fig. 5a) and in the small intestine homogenates (Fig. 5b). As expected, mMCPT-1 was significantly enhanced in serum and intestinal tissue in the allergic mice in comparison to the sham control mice. Although not significant, both pectin intervention approaches provided evidence for a slight reduction of mMCPT-1 level in the intestinal homogenate (Fig. 5b). Whereas the effect of pectin on serum derived mMCPT-1 in the therapeutic setting was negligible, mMCPT-1 was significantly suppressed when pectin was applied in the primary-preventive approach. Here, mMCPT-1 levels were diminished close to the baseline level of non-allergic mice (Fig. 5a).

### Pectin intervention suppressed manifestation of T_H_2-related antibody responses

**Fig. 6:**
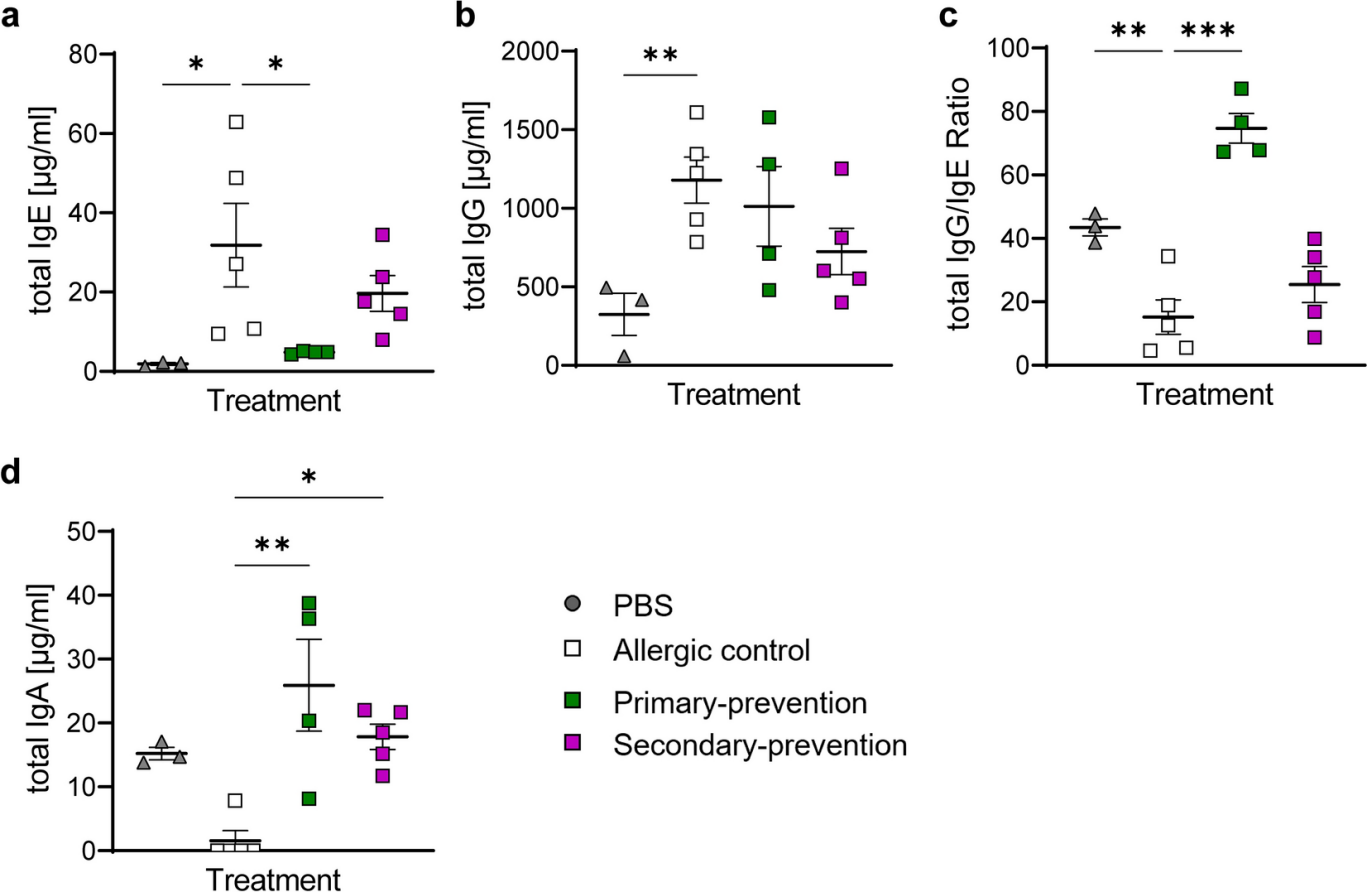
Immunoglobulin levels in serum. Levels of (**a**) total IgE, (**b**) total IgG and (**c**) total IgA were analyzed in the serum of the mice after provocation via ELISA. (**d**) Ratio of total IgG/IgE were analyzed. n = 3–5; *p < 0.05; **p < 0.01; ***p < 0.001, one-way ANOVA was used.

**Fig. 7:**
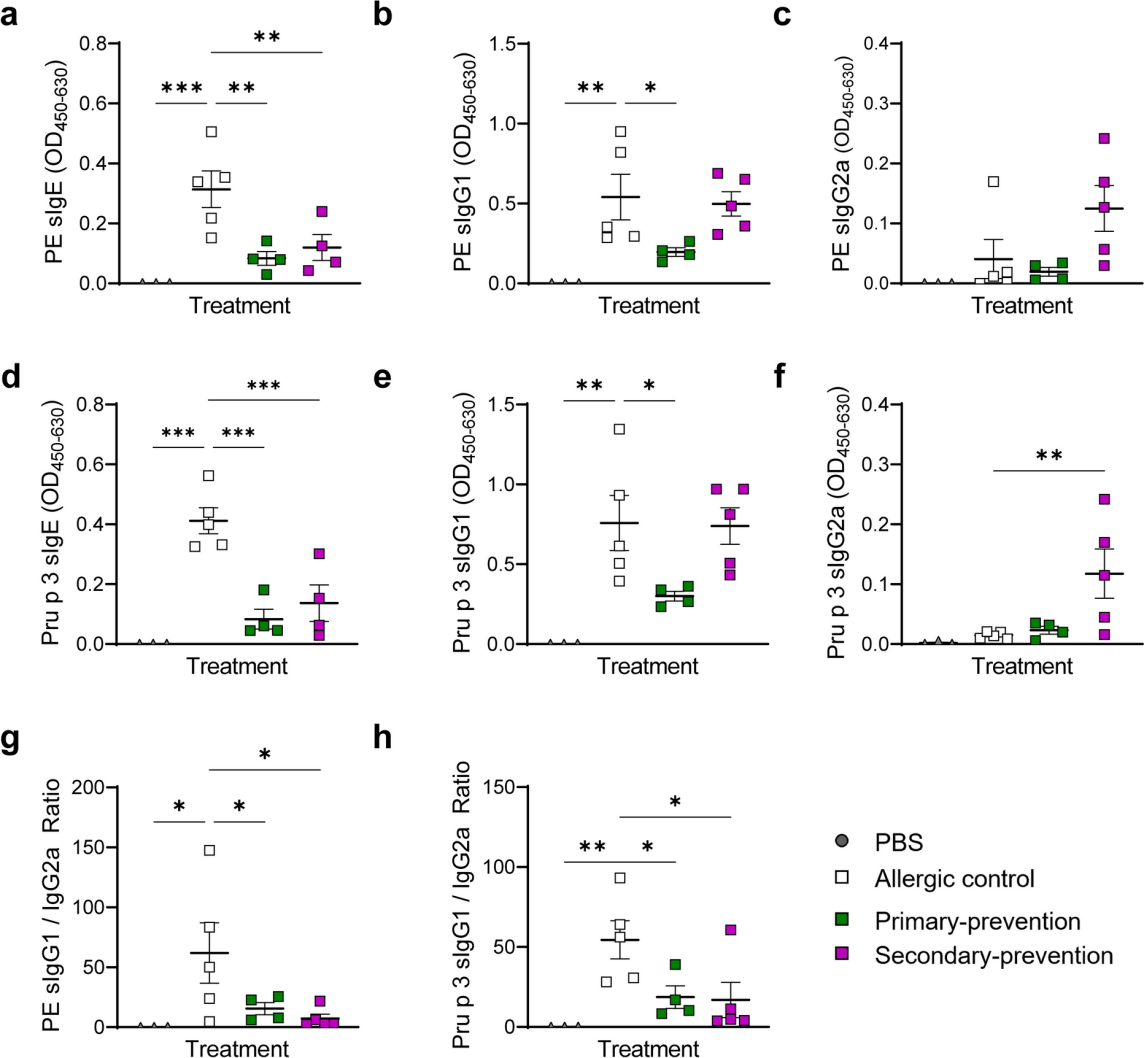
Pru p 3- and PE-specific antibody response in the serum. Levels of PE-specific (**a**) IgE, (**b**) IgG1 and (**c**) IgG2a, as well as Pru p 3-specific (**d**) IgE, (**e**) IgG1 and (**f**) IgG2a were analyzed in the serum of the mice after provocation via ELISA. Ratio of (**g**) PE-specific and (**h**) Pru p 3-specific IgG1 to IgG2a values were determined for baseline and final time point, respectively. n = 3–5; *p < 0.05; **p < 0.01; ***p < 0.001, one-way ANOVA was used.

Finally, the effect on the humoral immune response in the serum of the mice was evaluated, by the determination of the values of total and allergen-specific immunoglobulins (Fig. 6 and 7).

Interestingly, the primary-preventive approach significantly reduced the total IgE levels, while this effect was only moderate in the secondary-preventive approach (Fig. 6a) Pectin supplementation did not significantly alter levels of total IgG compared to the allergic control (Fig. 6b). However, when comparing the ratio of total IgG to total IgE, primary-preventive pectin supplementation significantly shifted the ratio towards IgG (Fig. 6c). Analysis of total IgA levels showed a strong decrease in the allergic control compared to the PBS treated mice, nevertheless, both pectin intervention approaches restored the total IgA levels to baseline (Fig. 6d).

Considering the modulatory effect of pectin intervention observed on the total immunoglobulin levels in serum and the modulatory effect of pectin on allergy-related immune response, allergen-specific antibody levels were determined in the serum of the mice after provocation (Fig. 7). The results indicate a strong increase of Pru p 3-specific IgE levels in the mice of the allergic control group that could be significantly reduced by pectin intervention. In comparison, only the primary-preventive intervention approach induced decreased levels of Pru p 3-specific IgG1, whereas the secondary-preventive intervention approach enhanced Pru p 3-specific IgG2a levels. Comparable effects could be observed for the PE-specific antibodies. In line with this, pectin interventions decreased the ratio of Pru p 3 and/or PE-specific IgG1/IgG2a compared to the allergic control.

## Discussion

Dietary fiber can regulate intestinal microbiome, can affect the immune responses in the gut and airways, and are important for the T_H_1/T_H_2 homeostasis^27^. Among dietary fibers pectin has been described to provide several beneficial properties by altering the GI-microbiota composition^15,18–20,32^.

Several studies have already shown a suppressive role on allergy manifestation by increasing the production of bacteria-derived SCFAs. SCFA like butyrate, propionate and acetate have been shown to mediate a reduced risk of allergic reactions^24,26,33–37^. The underlying mechanism of how dietary fibers can modulate the allergic asthma is reviewed by Verstegen and colleagues^23^. However, the role of pectin intervention on the manifestation of FA and the underlying immune response remains to be elucidated. Therefore, the present study aimed to explore the modulatory effect of pectin on gut microbiota composition, and its impact on the local and systemic immune reactions in a peach allergy mouse model.

Although pectin intervention was not sufficient to prevent the drop in the core body temperature of the mice after provocation, the clinical symptoms were partially reduced in the treated mice compared to the allergic controls. Intraperitoneal allergen provocation induced a drastic temperature drop of > 2 °C, a criterion for termination of the experiment, underlining the strong allergic response in the mouse model of peach allergy used in this study. However, systemic provocation does not replicate the natural intake of a food allergens via ingestion, where the allergens encounters the immune system via the GI-mucosa. The GI-tract being supposedly the location where pectin primarily impacts the immune system. Thus, it would be interesting to monitor the effect of pectin in a murine model of peach allergy with provocation via intragastric application and/or to increase pectin dosage. However, pectin ingestion has substantial effect in the GI-microbiota and general immune responses.

Interestingly, in the allergic control group the relative frequency of *Bacteroides spp.* was reduced upon allergic sensitization. In both, the primary-preventive and the secondary-preventive approach pectin intervention modulated the composition of the gut microbiota in an established allergic setting. This was evidenced by a strong shift towards *Bacteroides spp.* as early as 4 days after starting the pectin diet, resulting in higher frequencies of *Bacteroides spp.* (appr. 45–60%) than the non-allergic controls (appr. 15–40%). The results are in agreement with previous own data^30^ and independent report^21^. Members of the *Bacteroides* genus are well-known effective pectin-degraders due to the expression of carbohydrate-active enzymes (CAZymes)^38^. The generated pectin-derived oligosaccharides can be further utilized by bacteria to affect microbiota composition^39^. In addition, *Bacteroides spp.* are known to produce SCFAs as acetate, propionate and butyrate^40^, which are reported to dampen allergic airway T_H_2 responses, reduce inflammatory responses in vitro and in vivo^41,42^, and to the protection against development of experimental allergic airway inflammation by promoting the activation of Tregs and inhibiting T_H_2 response without triggering a T_H_1 response^43^. In line with this, a reduction of *Bacteroides spp.* in the intestinal microbiota has been associated with a higher risk of FA^26,44^. In contrast to the literature^45^, the frequency of the genus *Parabacteroides* was reduced after pectin intervention, which probably is a consequence of the efficient utilization of the pectin by *Bacteroides*, promoting its predominant abundance and reducing the diversity as indicated by the Chao 1 index. A similar reduction after pectin intervention was observed for the genus *Akkermansia*. The implications of this difference are still under discussion. *Akkermansia spp.* are considered to promote beneficial health effects^46^, whereas other publications have reported that *Akkermansia spp*. are pathobionts exacerbating the development of inflammation and food allergy in fiber-deprived mice by increasing the intestinal permeability^47^, or promoting the development of other pathobionts^48^. Despite *Blautia spp.* are known to produce SCFAs^49^ and is considered to have beneficial effects promoting intestinal health, reducing inflammation, metabolic diseases and antibacterial activity the frequency of *Blautia spp.* was not substantially enhanced after pectin supplementation, which does not confirm previous reports^50^.

The present study did not aim to analyze the role of SCFAs and other bacterial-derived metabolites, rather than to investigate the pectin-induced effects on the local and systemic immune response in relation to the microbiota under allergic conditions. Actually the production of bacteria-derived 2-methylbutyric acid in response to feeding of naïve mice with the same pectin has been reported in own previous study^30^.

Pectin intervention strongly affects the local cellular immune response in the small intestine, as observed by the variations in the frequency of immune cells in the intestinal lamina propria. Reduced frequencies of mast cells, macrophages, T cells, and CTLs were observed, particularly in the primary-preventive pectin intervention approach. In contrast, at the same time the frequency of B cells and CD4 + T cells was enhanced. Although the effects on TH1- and TH2-promoting cytokines in gastrointestinal tissue were not investigated in the study, the reduced mMCPT-1 levels provide some evidence of a reduced anti-inflammatory response. The results support protective effects of pectin supplementation on inflammatory responses and the manifestation of allergic reactions. Mast cell numbers are increased in IgE-dependent allergies which is correlated with the release of a variety of mediators as proteases, cytokines, lipid mediators and histamine, that lead to the recruitment of leukocytes, vasodilatation and characteristic inflammatory responses^51,52^. Mast cells in the intestinal mucosa are very well known effector cells in IgE-mediated FAs. Studies using mast cell-deficient mice have shown that a reduction of this cell type usually translates to a reduction in the allergic response and sensitization status^53^. In our study, pectin intervention reduced the frequency of mast cells in the small intestine of peach allergic mice, and in addition also reduced their activation status evidenced by the mMCPT levels. This supports the reduced development of clinical signs after provocation.

Remarkably, pectin intervention was also capable to modulate the humoral immune response. Under allergic condition mice showed enhanced allergen-specific IgE- and IgG1 values^52,54^, whereas total IgA response was reduced. In the same context, reduced IgA levels have been associated to a higher risk of allergic disease^54–56^. Interestingly, pectin intervention was able to reverse this effect, restoring the levels of specific and total IgE and IgA to the baseline level of non-allergic mice, while no clear effect was observed in the total IgG levels. However, when the ratio IgG/IgE was evaluated, a shift towards IgG was observed. Pectin has been shown to suppress IgE production in a human myeloma cell line in vitro^57^, and to significantly reduce the IgE levels in mice^58,59^. These reports are in line with the present study further supporting the hypothesis that pectin could be beneficial to reduce allergic reaction and downregulate the inflammatory response in the intestinal mucosa. As the isotype level of allergen-specific IgG1 was diminished while IgG2a was enhanced, the ratio of specific IgG1 to IgG2a was significantly decreased, suggesting a deviation towards a T_H_1 immune response^60^.

It remains unclear which structural features of pectin mediate the observed effect. Fermentation experiments showed that LMPs are fermented in vitro more efficiently, whereas HMPs exerts higher modulatory capacity on the gut microbiota composition^22,61^. Since different pectin subunits are degraded at different rates, structurally different pectins might exert distinct immune modulatory effects^22,62^. We can therefore only speculate if additional direct effect of pectin on epithelial and immune cells might as well play a role^15^. It is also known that pectin can also impact on the permeability of the gut and preserve the epithelial integrity^19^. Rhammnogalacturan-I enriched polysaccharides have been discussed to promote this effect^63^*.* However, it remains elusive which fine structure of pectins may have a beneficial effect on allergic inflammation.

In summary, this study showed that dietary pectin intervention shifted the gut microbiota composition towards *Bacteroides* which was strongly associated with a modulation of the local immune response analyzed by infiltrating cells in the lamina propria. Furthermore, food allergy-related humoral T_H_2 responses were significantly suppressed in the mice supplemented with pectin. Thus, these results suggest a beneficial role of pectin in the manifestation of FA shifting the immune response from T_H_2 towards T_H_1, favoring immune tolerance and providing anti-inflammatory properties.

## Materials and methods

### Pectin diet

Apple-derived pectin Herbapekt SF 50-LV, a HMP with a degree of esterification (DE) of 57%, galacturonic acid content of 52–61 mol%, and low molecular weight (MW) of 38–47 kDa was provided by Herbstreith & Fox GmbH & Co. KG (Neuenbürg, Germany). Food pellets for dietary intervention were prepared by ssniff Spezialdiäten GmbH (Soest, Germany) and contained 20% cellulose (control diet) or 15% pectin supplemented with 5% cellulose (Table S2).

### Animals

Female CBA/J mice (6–8 weeks old) were purchased from Charles River Deutschland GmbH and housed under specific pathogen-free conditions in the animal facility of the Paul-Ehrlich-Institut with free access to water and food. All animal experiments were performed in compliance with the German Animal Welfare Act and the. The authors complied with the ARRIVE guidelines. The study and protocols were reviewed by the animal welfare officer of the Paul-Ehrlich-Institut, and approved by the responsible authority, RP Darmstadt, Germany according the German animal protection law (approval number F107/2005).

### Pectin intervention in peach allergic mice

For pectin dietary intervention in peach allergic mice, sensitization and provocation was performed as described recently^64^ (Fig. 1). Briefly, mice were sensitized intraperitoneally (i.p.) with 200 µg peach peel extract (PE; in 200 µl; n = 5) or PBS (200 µl; n = 3) at d0, d7 and d12 using alum as adjuvant (1 mg per mouse). Afterwards, animals were exposed to 500 µg PE protein or PBS by oral gavage (i.g.) for three times in a two-days interval (d19, d21, d23). Provocation was performed by i.p. injection of 100 µg PE protein (in 200 µl) or PBS at d26, and symptom scores were recorded in a blinded manner (Table S1). Mice of the control groups (PBS or allergic control) were fed control diet starting two weeks before sensitization until the end of the experiment. Composition of the control diet (20% cellulose) and the pectin diet (15% pectin and 5% cellulose) is depicted in Table S2. Mice in the primary-preventive pectin intervention group were fed control diet for one week, followed by pectin diet starting 7 days before sensitization. In the secondary-preventive pectin intervention group, mice were fed control diet until start of the pectin diet after sensitization (d12). Body temperature and symptom score were monitored up to 30 min after each oral exposition and provocation. Feces was collected at day d-7, d-2, d12, d16, and d26. Mice were sacrified by euthanasia using Co2, sera and intestinal tissue were taken and stored at − 80 °C until use.

### Analysis of microbiome by metagenomic 16S rRNA gene sequencing

Bacterial DNA was extracted using the QIAamp PowerFecal Pro DNA Kit (Qiagen) and quantified using the QuantiFluor ONE dsDNA System on a Quantus™ Fluorometer (Promega). Amplification of 16S V3 and V4 region was performed by amplicon PCR using amplicon primers as described by Klindsworth et al.^65^, including Illumina adapter overhang sequence. Subsequently, DNA clean-up was performed and Illumina sequencing adapters were attached using the Nextera XT Index Kit and Index PCR. PCR clean-up was performed and final library was validated using the Agilent D1000 ScreenTape System (Agilent). Samples were adjusted to a final DNA concentration of 4 nM and paired-end sequencing was performed on an Illumina MiSeq benchtop sequencer (Illumina Inc., San Diego, USA) using 2 × 300 base, paired-end setup.

Processing of the sequencing data was performed by Qiime2 package^66^ using DADA2 (v.1.22.0)^67^ and Diversity (2022.8) plug-ins. Non-redundant representative sequences were taxonomically classified using the SILVA138 database^68^.

### Intestinal tissue histology and homogenates

Length of intestinal sections were determined, and longitudinal sections of small intestinal tissue (approximately 2 cm) were taken from the jejunum (9.5 cm distal to the duodenum) of CBA/J mice. The tissue sections were fixed in 4% formalin and embedded in paraffin. Sections of 5 μm thickness were prepared using microtome (Microm HM355S, Thermo Scientific) and stained with hematoxylin and eosin (H&E) for morphologic analysis.

Intestine homogenates were prepared as previously reported^69^. Jejunal tissue (10 cm length) was collected, Peyer’s patches were removed and the tissue was washed with cold PBS, cut in small pieces and subsequently frozen in liquid nitrogen. The frozen tissue was minced using mortar and pistil and the obtained powder was resuspended in 300 µl of cold PBS containing 1 × protease inhibitor (Merck KGaA, Darmstadt, Germany). Samples were centrifuged at 12,000×g for 20 min and supernatant was transferred to fresh tubes. Protein concentration was determined using BCA assay (Thermo Fisher Scientific) and adjusted to 5 mg/ml.

### Determination of antibody responses and mMCPT-1

For monitoring of antigen-specific antibody responses, major peach allergen Pru p 3, a non-specific lipid transfer protein (nsLTP), was purified from PE as described previously^70^. Natural (n) Pru p 3 (5 µg/ml) or PE (50 µg/ml) were coated on microtiter plates overnight at 4°C^64^. After blocking with 10% FCS in PBS for 2 h at RT, serum was added and incubated for 2 h at RT. Biotinylated anti-mouse IgE (R35-118; BD Biosciences) antibody was incubated for 1 h at RT, followed by 30 min incubation of HRP-labeled streptavidin. For detection of antigen-specific IgG1 (sIgG1) and sIgG2a, HRP-conjugated goat anti-mouse IgG1 (Thermo Fisher Scientific) or HRP-conjugated rabbit anti-mouse IgG2a (Thermo Fisher Scientific) were used. Antigen-specific antibodies were detected by addition of TMB-substrate followed by measurement of the absorbance at 450_nm_. Detection of total IgE, total IgG and total IgA was performed using commercial ELISA kits according to the manufacturer’s instruction (Thermo Fisher Scientific). In addition, mouse mast cell protease-1 (mMCPT-1) was quantified in serum and intestinal homogenates using commercial ELISA kits according to the manufacturer’s instruction (Thermo Fisher Scientific, Darmstadt, Germany).

### Preparation of lamina propria tissue

The lamina propria dissociation was performed following an adapted protocol from Weigmann et al.^71^. Briefly, small intestines were harvested, fat tissue and Peyer’s patches were removed. The intestines were washed with cold PBS, opened longitudinally and cut in 1 cm pieces. The samples were further washed in 1 × Hank’s Balanced Salt Solution (HBSS) containing 5 mM dithiothreitol (DTT) at 37 °C for 20 min. Intestine pieces were subsequently passed through a 100 µm cell strainer and incubated in pre-digestion solution (1 × HBSS containing 5 mM EDTA and 10 mM HEPES) for 20 min at 37 °C using slow rotation. The samples were again passed through a 100 µm cell strainer followed by repeated incubation in pre-digestion solution. Afterwards, the intestine pieces were washed using 1 × HBSS containing 10 mM HEPES, passed through a cell strainer (100 µm) and incubated in digestion solution (0.5 mg/ml Collagenase D; 0.5 mg/ml DNase I; 1 mg/ml Dispase II in PBS) for 20 min at 37 °C using slow rotation. The samples were subsequently passed through a 40 µm cell strainer and the flow through was collected in cold FCS. The isolated cells were repeatedly washed in cold PBS, counted and used for further analysis by FACS.

### FACS analysis of lamina propria cells

Single cell suspensions of lamina propria cells underwent Fc block with anti-CD16/32 (Clone 93*;* eBioscience, Frankfurt am Main, Germany) followed by staining with extracellular antibodies, viability dye (Thermo Fisher Scientific) and if applicable nuclear staining (true-nuclear, BioLegend). Antibodies and gating strategies are depicted in Table S3 and Figures S6 and S7, respectively. Data were acquired using FACS Symphony (BD Biosciences, Heidelberg, Germany) and analyzed via FlowJo (version 10.0.8r1; BD Biosciences).

### Statistical analysis

The results are shown as combined data from two independent mouse experiments conducted under the same experimental settings. The results are represented as means ± SEM, and the data were statistically evaluated by Mann–Whitney U test or ANOVA (α = 0.05) using Graph Pad Prism version 9.5.0 https://www.graphpad.com. For statistical analysis of the sequencing data, the processed 16S V4 rRNA amplicon high throughput sequencing data was analyzed using QIIME 2 software version 2022.8.3 https://qiime2.org/.

## Supplementary Information

Supplementary Information.

## Data Availability

The datasets generated and analyzed during the current study are available in the European Nucleotide Archive repository (https://www.ebi.ac.uk/ena/browser/home), accession PRJEB81529.

## Abbreviations

FA: Food allergy
PE: Peach peel extract
i.p.: Intraperitoneal
mMCPT-1: Mouse mast cell protease-1
OIT: Oral immunotherapy
SIT: Sublingual immunotherapy
GI: Gastrointestinal
SCFA: Short chain fatty acids
HMP: High-methoxyl pectin
LMP: Low-methoxyl pectin
DE: Degree of esterification
i.g.: Intragastric gavage
HBSS: Hank’s Balanced Salt Solution
DTT: Dithiothreitol
cDCs: Conventional dendritic cells
