# Supplementary material for: Dietary fiber pectin alters the gut microbiota and diminishes the inflammatory immune responses in an experimental peach allergy mouse model
Source: Sci Rep. 2024 Dec 16;14:30503. doi: 10.1038/s41598-024-82210-3 (PMC11649683; doi:10.1038/s41598-024-82210-3)

Table S1: Symptom score for quantification of allergy-related symptoms

Allergy-related symptoms were evaluated in a blinded approach after oral boost and provocation according to the parameters indicated in the symptom score sheet.

Symptom Score	0	1	2
**Behaviour**	normal	calm	apathetic
**Fur**	normal	slightly ruffled	ruffled
**Stool**	normal	Soft	with mucus
**Body temp.**	$\Delta < -1$°C	$\Delta < -1$ to -2°C	$\Delta > -2$°C

Table S2: Composition of fiber adjusted diet

Food pellets ingredients of the control diet (20% cellulose) or the pectin diet (15% pectin plus 5% cellulose).

1) Calculated with 94 % fiber, 2) Pectin provided by the customer: Herbapekt, 3) Physiological fuel value

Ingredient	U	**20% Cellulose** AIN93G mod. S8144-E730	**15% Pectin** 5% Cellul. AIN93G S8144-E734 / E738
Casein	%	20.0000	20.0000
L-Cystine	%	0.3000	0.3000
Cellulose powder	%	20.0000	5.0000
Pectin 1)2)	%	---	15.0000
Corn starch	%	26.7486	26.7486
Maltodextrin	%	13.2000	13.2000
Sucrose	%	10.0000	10.0000
Vitamin premix	%	1.0000	1.0000
Mineral premix	%	3.5000	3.5000
tBHQ	%	0.0014	0.0014
Choline bitartrate	%	0.2500	0.2500
Soybean oil	%	5.0000	5.0000
**Proximate contents**			
Crude protein	%	17.6	17.6
Crude fat	%	5.1	5.1
Crude fiber	%	19.9	19.1
Crude ash	%	3.2	3.2
Starch	%	25.7	25.7
Dextrin	%	13.0	13.0
Sugar	%	11.2	11.2
**Energy (Atwater) 3) MJ/kg**		**13.4**	**13.4**
kcal% Protein		22	22
kcal% Fat		15	15
kcal% Carbohydrates		63	63

Table S3: Antibodies used for flow cytometry

Used antibodies and specifications for flow cytometry

Target	Label	Clone	Company
**CD45**	FITC	30-F11	BioLegend
**CD11c**	BV605	N418	BioLegend
**CD11b**	BV421	M1/70	BioLegend
**MHCII**	BV510	M5/114.15.2	BioLegend
**CD64**	PECy7	X54-5/7.1	BioLegend
**CD117**	BV711	2B8	BioLegend
**Ly6G**	Alexa647	1A8	BioLegend
**SiglecF**	PE	S17007L	BioLegend
**CD3**	BV421	17A2	BioLegend
**CD4**	PECy7	GK1.5	BioLegend
**CD8a**	BF510	53-6.7	BioLegend
**CD19**	PE-BV605	6D5	BioLegend
**CD25**	BV711	PC61	BioLegend
**FoxP3**	PE	150D	BioLegend

Figure S1: Monitoring of body weight gain and food intake

(a) Total body weight gain (g/mouse) and (b) food intake (g/mouse) over the period (26 days) of the experiment are depicted. n = 3-5

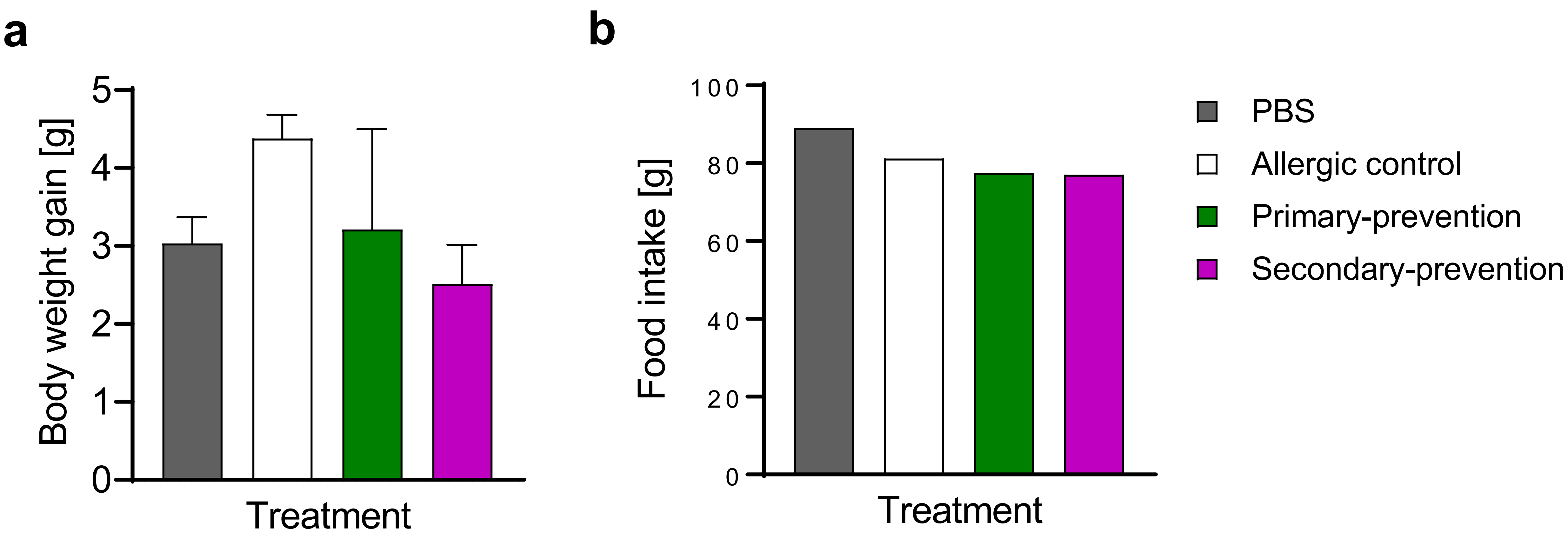

Figure S2: Intestinal size and histological analysis

(a) Length of small intestine, (b) large intestine and (c) caecum were measured after the provocation of the mice. n = 3-5; *$p < 0.05$; **$p < 0.01$; ***$p < 0.001$

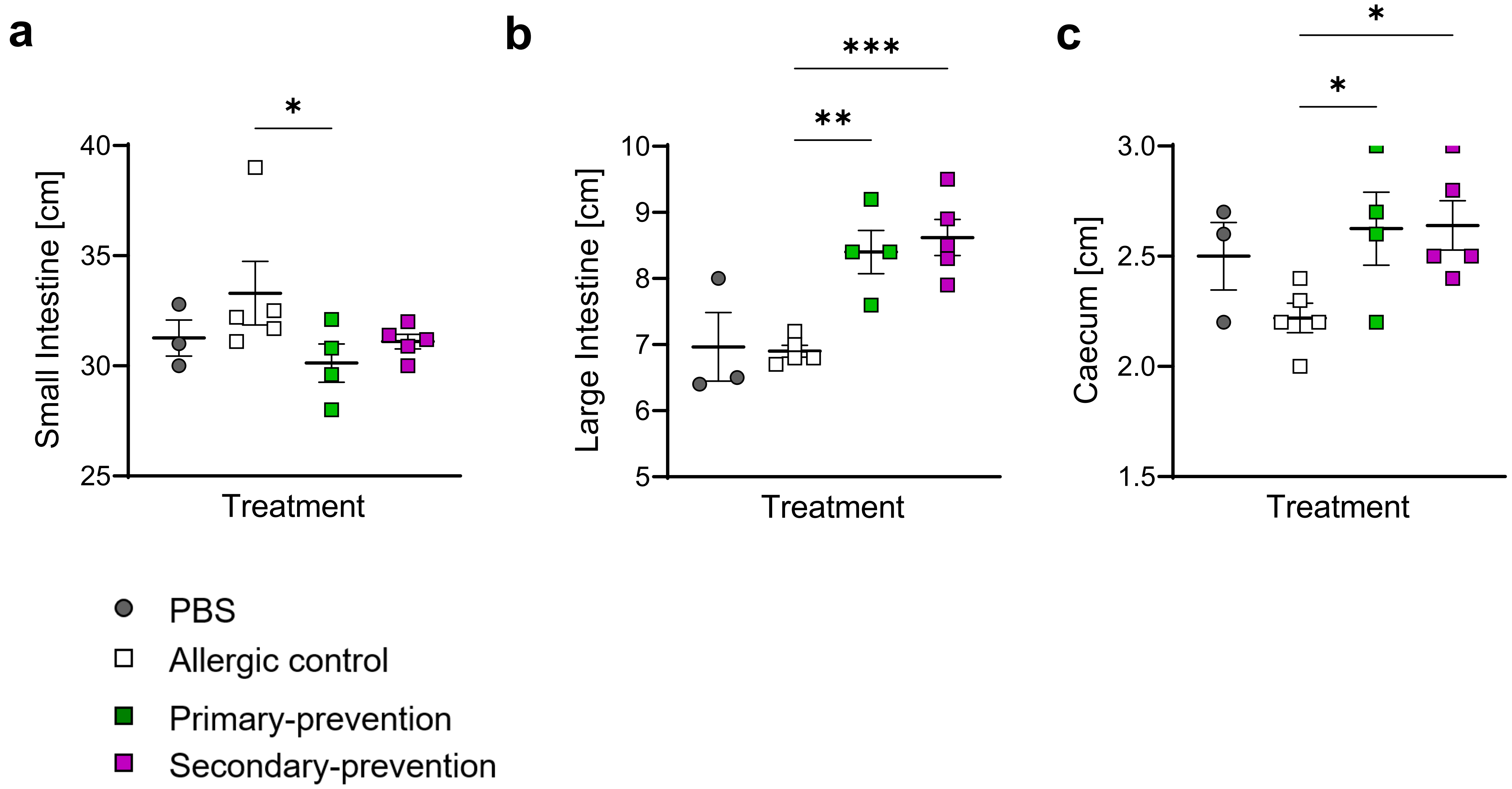

Figure S2

Figure S3: Relative frequency of Bacteroidaceae and Akkermansiaceae

Time dependent alteration of the relative frequency of Bacteroidaceae and Akkermansiaceae are depicted seperately for each group.

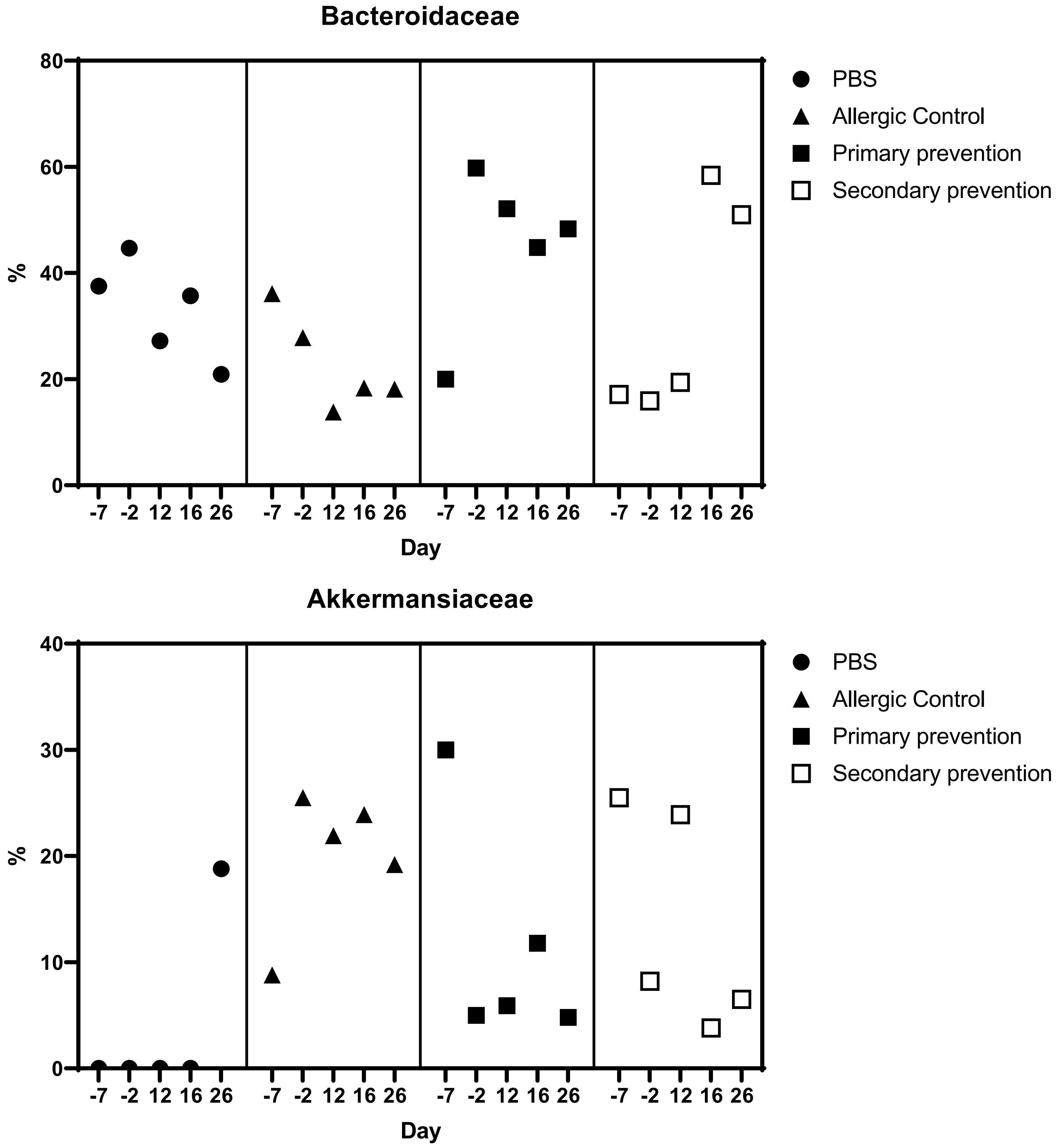

Table S4: Microbiota analysis

16S RNA analysis of 39 bacterial strains in each group (PBS control, peach allergy, prophylaxis = primary-prevention and therapy = secondary-prevention) depicted by number of reads (A) and relative frequency (%) in B. Results for Bacteroidaceae and Akkermansiaceae are highlighted.

index	Tannerellaceae	Bacteroidaceae	Akkermansiaceae	Lachnospiraceae	Enterobacteriaceae	Erysipelotrichaceae	Enterococcaceae	Burkholderiaceae	Ruminococcaceae	Rikenellaceae	Rhodospirillales	Desulfovibrionaceae	Muribaculaceae	Lactobacillaceae	Atopobiaceae	Streptococcaceae	Deferribacteraceae	Eggerthellaceae	Peptococcaceae
PBS_-7	38607	146691	143	102639	63	6606	39	3179	18462	24369	1527	3073	37819	1074	0	1255	111	3334	828
PBS_-2	81074	172248	20	49518	34356	13960	827	19160	9049	428	756	1199	87	55	0	471	172	1229	279
PBS_12	129340	137431	46	93790	53635	18673	20912	18027	12841	6633	1060	709	7501	708	0	614	76	1248	369
PBS_16	123924	206133	24	131129	27990	37280	2360	20173	14674	4152	1631	954	2664	503	15	747	143	1815	342
PBS_26	115084	128131	115653	47420	128262	4781	43583	16261	10029	2497	230	606	116	146	16	185	104	473	130
Peach_-7	45172	175961	42809	114532	9528	38083	603	23386	11638	4483	4923	3595	4839	244	0	1144	1537	2518	278
Peach_-2	132791	228614	209979	111184	31801	16692	27979	28690	14133	5762	7500	2128	2079	275	0	652	114	1048	209
Peach_12	173566	111152	176546	84282	91127	9621	102209	23010	12689	5655	1743	2526	5738	782	194	1247	1499	1879	997
Peach_16	224668	196830	257923	167372	57923	35592	15784	31880	34321	16435	3332	8263	17799	130	2424	458	978	1958	1504
Peach_26	183964	141807	150635	71795	137675	20450	18480	26281	14270	5387	3802	972	3817	166	3406	327	26	474	560
Therapy_-7	123643	94572	141334	95294	797	8528	4591	12487	21451	18908	3447	6333	14767	778	0	2716	715	1662	486
Therapy_-2	189384	93399	48057	25005	56614	15943	111848	18403	15353	1153	57	4140	982	89	0	622	4135	156	239
Therapy_12	351188	218741	268640	133700	13487	17641	6812	36015	32212	14568	2047	7642	12415	108	291	1848	1766	3703	1555
Therapy_16	9964	223321	14379	80424	14523	22655	7836	144	6476	0	0	0	17	1656	384	128	0	414	0
Therapy_26	49193	497489	63722	142010	139303	46071	8729	7342	9870	0	0	109	33	7619	1841	235	154	565	188
Profilaxis_-7	92645	143053	214273	105286	12125	28901	7815	16688	23196	14996	14785	4138	29230	450	0	692	711	1959	1026
Profilaxis_-2	20380	459012	38222	115618	69973	37136	7908	3077	11288	0	0	0	0	2049	2639	154	0	444	0
Profilaxis_12	29516	298601	33988	78933	87741	24763	3854	6081	4684	0	0	0	0	2439	1749	134	5	95	5
Profilaxis_16	57697	348523	92093	72554	140608	35468	4621	12848	7077	0	0	0	0	2601	3307	114	33	325	9
Profilaxis_26	30714	218374	21486	53557	97686	16662	2766	4224	3473	0	0	0	0	2241	507	82	0	133	0

index	Clostridiales	Marinifilaceae	Mollicutes	Defluviitaleaceae	Christensenellaceae	Mollicutes RF39	Prevotellaceae	Saccharimonadaceae	Bacteria unknown	Eubacteriaceae	Gastranaerophilales	Clostridiales vadinBB60	Unassigned	Betaproteobacteriales	Clostridiales_uncultured	Corynebacteriaceae	Peptostreptococcaceae	Clostridiaceae 1	Pseudomonadaceae	Clostridiales_	Summe
PBS_-7	1213	0	131	0	111	0	125	35	0	23	0	0	0	0	7	0	0	0	0	0	391464
PBS_-2	342	0	0	0	0	0	0	0	0	0	0	0	0	0	0	0	0	0	0	0	385230
PBS_12	554	420	124	0	0	0	0	0	0	0	0	0	0	0	0	0	0	0	0	0	504711
PBS_16	539	0	24	0	9	0	0	0	5	0	0	0	0	0	0	0	0	0	0	0	577230
PBS_26	153	0	0	0	0	0	0	0	0	0	0	0	10	0	0	0	0	0	0	0	613870
Peach_-7	1908	45	122	3	0	0	0	0	5	0	0	6	0	0	6	0	0	0	0	0	487368
Peach_-2	681	0	0	0	0	0	0	0	0	0	0	0	0	0	0	0	0	0	0	0	822311
Peach_12	287	0	49	0	21	0	0	0	0	0	0	0	0	0	0	0	0	2	0	0	806821
Peach_16	2382	0	40	0	63	0	0	0	0	0	35	0	0	0	0	0	2	0	0	0	1078096
Peach_26	240	0	0	0	0	0	0	0	0	0	0	0	0	0	0	0	0	0	0	0	784534
Therapy_-7	646	187	0	0	52	0	0	60	15	0	4	12	0	0	0	0	3	0	0	2	553490
Therapy_-2	227	0	0	0	11	0	0	0	14	0	0	0	0	0	0	0	0	0	0	0	585831
Therapy_12	412	0	0	12	64	0	0	0	7	0	0	0	0	7	0	0	0	0	0	0	1124881
Therapy_16	21	0	0	0	0	0	0	0	0	0	0	0	0	0	0	0	0	0	0	0	382342
Therapy_26	265	0	0	92	2	0	0	0	6	0	0	0	0	0	0	0	0	0	0	0	974838
Profilaxis_-7	1376	0	55	242	129	203	36	0	2	11	0	11	0	0	0	0	0	0	0	0	714034
Profilaxis_-2	93	0	0	0	8	0	0	0	18	0	0	0	0	0	0	0	0	0	2	0	768021
Profilaxis_12	33	0	0	44	21	0	0	0	0	4	0	0	0	0	0	3	0	0	0	0	572693
Profilaxis_16	201	0	0	90	71	0	0	0	0	6	0	0	0	0	0	0	0	0	0	0	778246
Profilaxis_26	0	0	0	0	0	0	0	0	0	0	0	0	0	0	0	0	0	0	0	0	451905

index	Tannerellaceae	Bacteroidaceae	Akkermansiaceae	Lachnospiraceae	Enterobacteriaceae	Erysipelotrichaceae	Enterococcaceae	Burkholderiaceae	Ruminococcaceae	Rikenellaceae	Rhodospirillales	Desulfovibrionaceae	Muribaculaceae	Lactobacillaceae	Atopobiaceae	Streptococcaceae	Deferribacteraceae	Eggerthellaceae	Peptococcaceae	Clostridiales
PBS_-7	9,9%	37,5%	0,0%	26,2%	0,0%	1,7%	0,0%	0,8%	4,7%	6,2%	0,4%	0,8%	9,7%	0,3%	0,0%	0,3%	0,0%	0,9%	0,2%	0,3%
PBS_-2	21,0%	44,7%	0,0%	12,9%	8,9%	3,6%	0,2%	5,0%	2,3%	0,1%	0,2%	0,3%	0,0%	0,0%	0,0%	0,1%	0,0%	0,3%	0,1%	0,1%
PBS_12	25,6%	27,2%	0,0%	18,6%	10,6%	3,7%	4,1%	3,6%	2,5%	1,3%	0,2%	0,1%	1,5%	0,1%	0,0%	0,1%	0,0%	0,2%	0,1%	0,1%
PBS_16	21,5%	35,7%	0,0%	22,7%	4,8%	6,5%	0,4%	3,5%	2,5%	0,7%	0,3%	0,2%	0,5%	0,1%	0,0%	0,1%	0,0%	0,3%	0,1%	0,1%
PBS_26	18,7%	20,9%	18,8%	7,7%	20,9%	0,8%	7,1%	2,6%	1,6%	0,4%	0,0%	0,1%	0,0%	0,0%	0,0%	0,0%	0,0%	0,1%	0,0%	0,0%
Peach_-7	9,3%	36,1%	8,8%	23,5%	2,0%	7,8%	0,1%	4,8%	2,4%	0,9%	1,0%	0,7%	1,0%	0,1%	0,0%	0,2%	0,3%	0,5%	0,1%	0,4%
Peach_-2	16,1%	27,8%	25,5%	13,5%	3,9%	2,0%	3,4%	3,5%	1,7%	0,7%	0,9%	0,3%	0,3%	0,0%	0,0%	0,1%	0,0%	0,1%	0,0%	0,1%
Peach_12	21,5%	13,8%	21,9%	10,4%	11,3%	1,2%	12,7%	2,9%	1,6%	0,7%	0,2%	0,3%	0,7%	0,1%	0,0%	0,2%	0,2%	0,2%	0,1%	0,0%
Peach_16	20,8%	18,3%	23,9%	15,5%	5,4%	3,3%	1,5%	3,0%	3,2%	1,5%	0,3%	0,8%	1,7%	0,0%	0,2%	0,0%	0,1%	0,2%	0,1%	0,2%
Peach_26	23,4%	18,1%	19,2%	9,2%	17,5%	2,6%	2,4%	3,3%	1,8%	0,7%	0,5%	0,1%	0,5%	0,0%	0,4%	0,0%	0,0%	0,1%	0,1%	0,0%
Therapy_-7	22,3%	17,1%	25,5%	17,2%	0,1%	1,5%	0,8%	2,3%	3,9%	3,4%	0,6%	1,1%	2,7%	0,1%	0,0%	0,5%	0,1%	0,3%	0,1%	0,1%
Therapy_-2	32,3%	15,9%	8,2%	4,3%	9,7%	2,7%	19,1%	3,1%	2,6%	0,2%	0,0%	0,7%	0,2%	0,0%	0,0%	0,1%	0,7%	0,0%	0,0%	0,0%
Therapy_12	31,2%	19,4%	23,9%	11,9%	1,2%	1,6%	0,6%	3,2%	2,9%	1,3%	0,2%	0,7%	1,1%	0,0%	0,0%	0,2%	0,2%	0,3%	0,1%	0,0%
Therapy_16	2,6%	58,4%	3,8%	21,0%	3,8%	5,9%	2,0%	0,0%	1,7%	0,0%	0,0%	0,0%	0,0%	0,4%	0,1%	0,0%	0,0%	0,1%	0,0%	0,0%
Therapy_26	5,0%	51,0%	6,5%	14,6%	14,3%	4,7%	0,9%	0,8%	1,0%	0,0%	0,0%	0,0%	0,0%	0,8%	0,2%	0,0%	0,0%	0,1%	0,0%	0,0%
Profilaxis_-7	13,0%	20,0%	30,0%	14,7%	1,7%	4,0%	1,1%	2,3%	3,2%	2,1%	2,1%	0,6%	4,1%	0,1%	0,0%	0,1%	0,1%	0,3%	0,1%	0,2%
Profilaxis_-2	2,7%	59,8%	5,0%	15,1%	9,1%	4,8%	1,0%	0,4%	1,5%	0,0%	0,0%	0,0%	0,0%	0,3%	0,3%	0,0%	0,0%	0,1%	0,0%	0,0%
Profilaxis_12	5,2%	52,1%	5,9%	13,8%	15,3%	4,3%	0,7%	1,1%	0,8%	0,0%	0,0%	0,0%	0,0%	0,4%	0,3%	0,0%	0,0%	0,0%	0,0%	0,0%
Profilaxis_16	7,4%	44,8%	11,8%	9,3%	18,1%	4,6%	0,6%	1,7%	0,9%	0,0%	0,0%	0,0%	0,0%	0,3%	0,4%	0,0%	0,0%	0,0%	0,0%	0,0%
Profilaxis_26	6,8%	48,3%	4,8%	11,9%	21,6%	3,7%	0,6%	0,9%	0,8%	0,0%	0,0%	0,0%	0,0%	0,5%	0,1%	0,0%	0,0%	0,0%	0,0%	0,0%

index	Marinifilaceae	Mollicutes	Defluviitaleaceae	Christensenellaceae	Mollicutes RF39	Prevotellaceae	Saccharimonadaceae	Bacteria unknown	Eubacteriaceae	Gastranaerophilales	Clostridiales vadinBB60	Unassigned	Betaproteobacteriales	Clostridiales_uncultured	Corynebacteriaceae	Peptostreptococcaceae	Clostridiaceae 1	Pseudomonadaceae	Clostridiales_
PBS_-7	0,0%	0,0%	0,0%	0,0%	0,0%	0,0%	0,0%	0,0%	0,0%	0,0%	0,0%	0,0%	0,0%	0,0%	0,0%	0,0%	0,0%	0,0%	0,0%
PBS_-2	0,0%	0,0%	0,0%	0,0%	0,0%	0,0%	0,0%	0,0%	0,0%	0,0%	0,0%	0,0%	0,0%	0,0%	0,0%	0,0%	0,0%	0,0%	0,0%
PBS_12	0,1%	0,0%	0,0%	0,0%	0,0%	0,0%	0,0%	0,0%	0,0%	0,0%	0,0%	0,0%	0,0%	0,0%	0,0%	0,0%	0,0%	0,0%	0,0%
PBS_16	0,0%	0,0%	0,0%	0,0%	0,0%	0,0%	0,0%	0,0%	0,0%	0,0%	0,0%	0,0%	0,0%	0,0%	0,0%	0,0%	0,0%	0,0%	0,0%
PBS_26	0,0%	0,0%	0,0%	0,0%	0,0%	0,0%	0,0%	0,0%	0,0%	0,0%	0,0%	0,0%	0,0%	0,0%	0,0%	0,0%	0,0%	0,0%	0,0%
Peach_-7	0,0%	0,0%	0,0%	0,0%	0,0%	0,0%	0,0%	0,0%	0,0%	0,0%	0,0%	0,0%	0,0%	0,0%	0,0%	0,0%	0,0%	0,0%	0,0%
Peach_-2	0,0%	0,0%	0,0%	0,0%	0,0%	0,0%	0,0%	0,0%	0,0%	0,0%	0,0%	0,0%	0,0%	0,0%	0,0%	0,0%	0,0%	0,0%	0,0%
Peach_12	0,0%	0,0%	0,0%	0,0%	0,0%	0,0%	0,0%	0,0%	0,0%	0,0%	0,0%	0,0%	0,0%	0,0%	0,0%	0,0%	0,0%	0,0%	0,0%
Peach_16	0,0%	0,0%	0,0%	0,0%	0,0%	0,0%	0,0%	0,0%	0,0%	0,0%	0,0%	0,0%	0,0%	0,0%	0,0%	0,0%	0,0%	0,0%	0,0%
Peach_26	0,0%	0,0%	0,0%	0,0%	0,0%	0,0%	0,0%	0,0%	0,0%	0,0%	0,0%	0,0%	0,0%	0,0%	0,0%	0,0%	0,0%	0,0%	0,0%
Therapy_-7	0,0%	0,0%	0,0%	0,0%	0,0%	0,0%	0,0%	0,0%	0,0%	0,0%	0,0%	0,0%	0,0%	0,0%	0,0%	0,0%	0,0%	0,0%	0,0%
Therapy_-2	0,0%	0,0%	0,0%	0,0%	0,0%	0,0%	0,0%	0,0%	0,0%	0,0%	0,0%	0,0%	0,0%	0,0%	0,0%	0,0%	0,0%	0,0%	0,0%
Therapy_12	0,0%	0,0%	0,0%	0,0%	0,0%	0,0%	0,0%	0,0%	0,0%	0,0%	0,0%	0,0%	0,0%	0,0%	0,0%	0,0%	0,0%	0,0%	0,0%
Therapy_16	0,0%	0,0%	0,0%	0,0%	0,0%	0,0%	0,0%	0,0%	0,0%	0,0%	0,0%	0,0%	0,0%	0,0%	0,0%	0,0%	0,0%	0,0%	0,0%
Therapy_26	0,0%	0,0%	0,0%	0,0%	0,0%	0,0%	0,0%	0,0%	0,0%	0,0%	0,0%	0,0%	0,0%	0,0%	0,0%	0,0%	0,0%	0,0%	0,0%
Profilaxis_-7	0,0%	0,0%	0,0%	0,0%	0,0%	0,0%	0,0%	0,0%	0,0%	0,0%	0,0%	0,0%	0,0%	0,0%	0,0%	0,0%	0,0%	0,0%	0,0%
Profilaxis_-2	0,0%	0,0%	0,0%	0,0%	0,0%	0,0%	0,0%	0,0%	0,0%	0,0%	0,0%	0,0%	0,0%	0,0%	0,0%	0,0%	0,0%	0,0%	0,0%
Profilaxis_12	0,0%	0,0%	0,0%	0,0%	0,0%	0,0%	0,0%	0,0%	0,0%	0,0%	0,0%	0,0%	0,0%	0,0%	0,0%	0,0%	0,0%	0,0%	0,0%
Profilaxis_16	0,0%	0,0%	0,0%	0,0%	0,0%	0,0%	0,0%	0,0%	0,0%	0,0%	0,0%	0,0%	0,0%	0,0%	0,0%	0,0%	0,0%	0,0%	0,0%
Profilaxis_26	0,0%	0,0%	0,0%	0,0%	0,0%	0,0%	0,0%	0,0%	0,0%	0,0%	0,0%	0,0%	0,0%	0,0%	0,0%	0,0%	0,0%	0,0%	0,0%

Figure S4: Relative frequency of microbiota from fecal samples

Time dependent alteration of the relative frequency of gastrointestinal microbiota for individual mice of each group is shown (%).

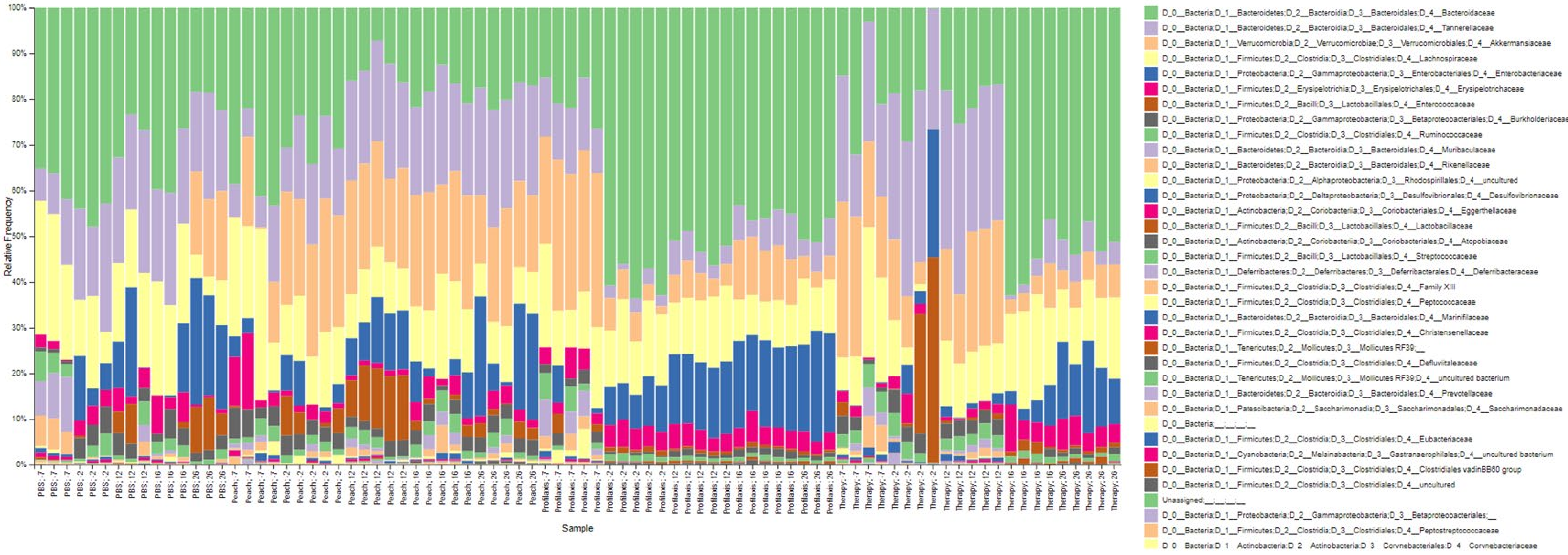

Figure S5: Histological analysis of small intestine

Histological changes in the jejunum were examined by H&E staining.

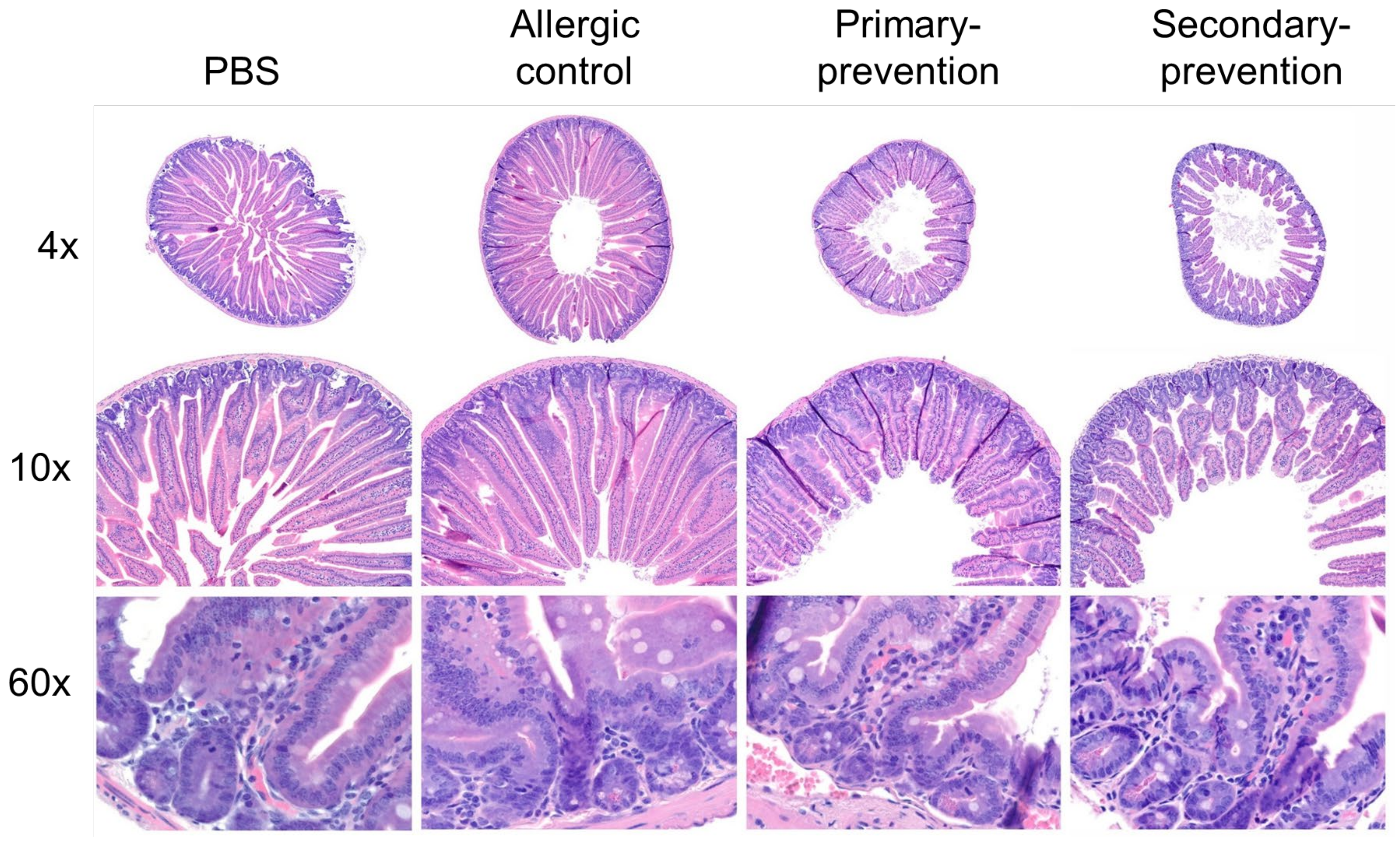

Figure S6: Gating strategy for T cell and B cell panel

Live lymphocytes were gated for FSC, SSC characteristics, cleaned up from doublets and identified as CD45+ live population. Among these, B cells were identified as CD19+CD3- and T cells as CD19-CD3+ cell population. T cells were further discriminated for CD8 of CD4 expression and Tregs were identified as Foxp3+ population among CD4 cells.

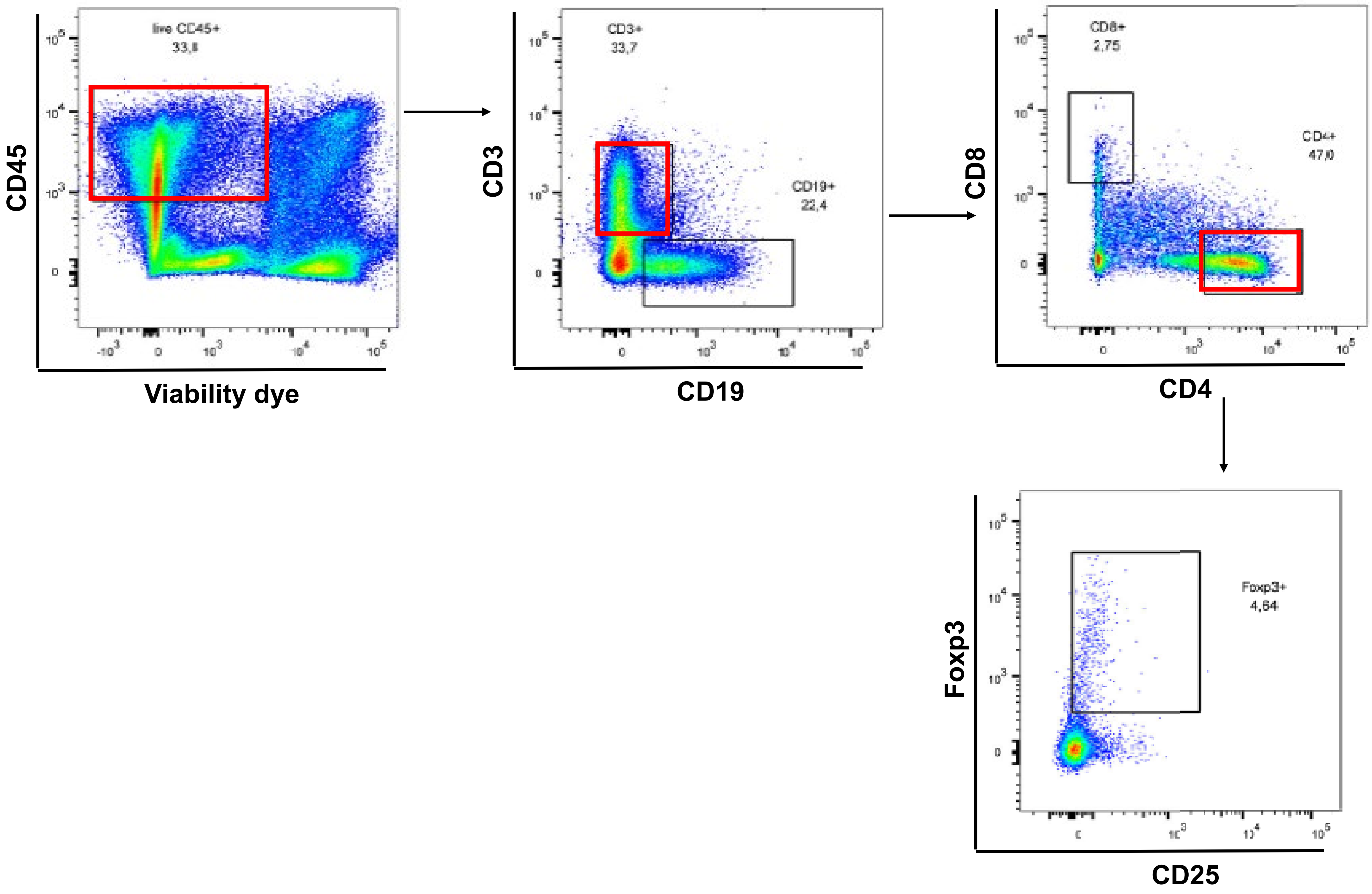

Figure S7: Gating strategy for granulocyte panel

Live lymphocytes were gated for FSC, SSC characteristics, cleaned up from doublets and identified as CD45+ live population. Among these, CD11b+ and CD11b- populations were gated and eosinophils were identified as SiglecF+Ly6G- among the CD11b+ population. Mast cells were gated as CD117+SiglecF- among the CD11b- population. Additionally, MHCII+ cells were identified among CD45+ live cells and subsequently analyzed for CD11c+CD64- conventional dendritic cells (cDCs) and CD11c+CD64+ macrophages.

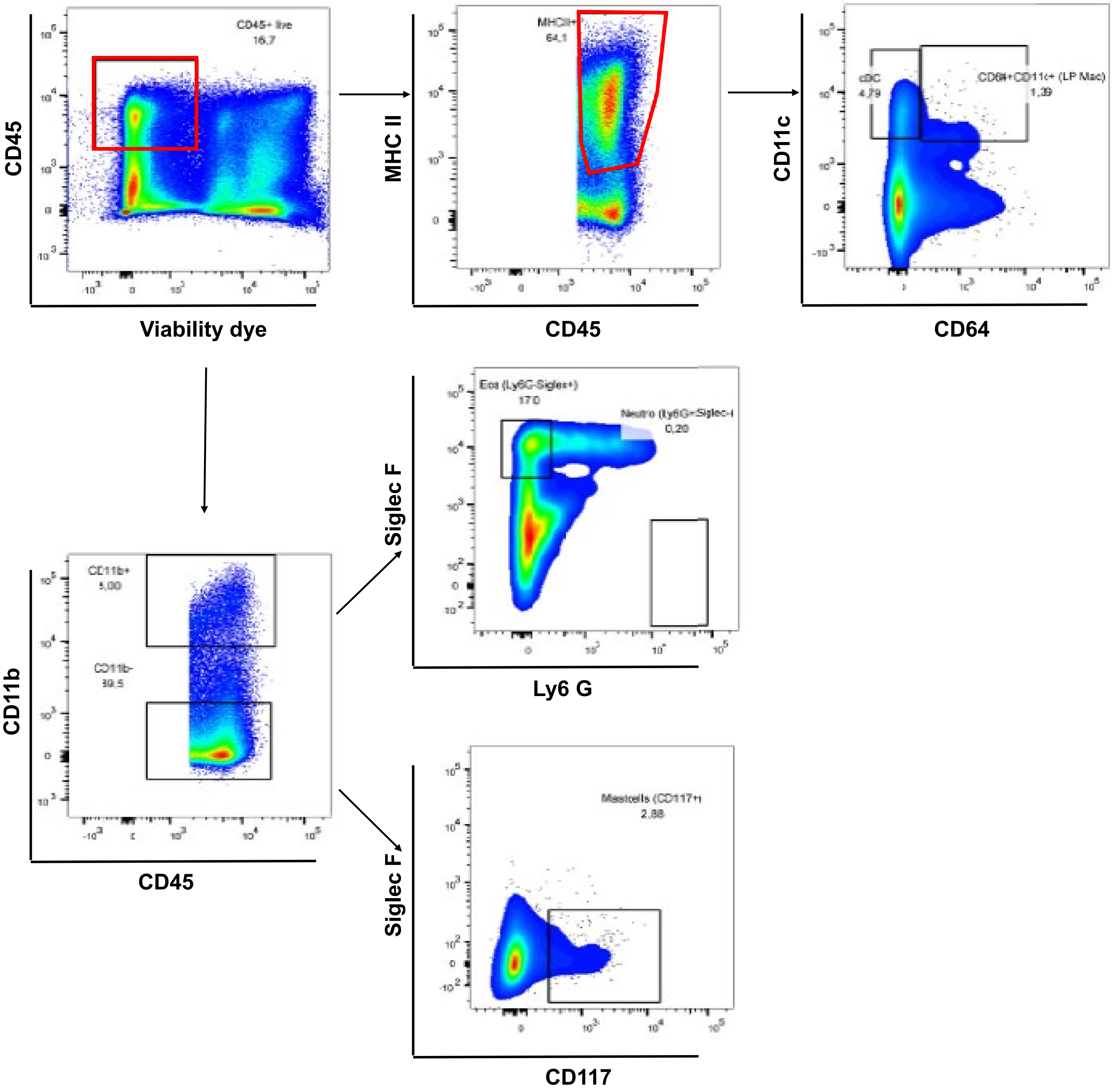

Supplement: Supplementary file 1 — Supplementary Information. [file 41598_2024_82210_MOESM1_ESM.pdf]
